# High-Up: A Remote Reservoir of Microbial Extremophiles in Central Andean Wetlands

**DOI:** 10.3389/fmicb.2015.01404

**Published:** 2015-12-16

**Authors:** Virginia H. Albarracín, Daniel Kurth, Omar F. Ordoñez, Carolina Belfiore, Eduardo Luccini, Graciela M. Salum, Ruben D. Piacentini, María E. Farías

**Affiliations:** ^1^Laboratorio de Investigaciones Microbiológicas de Lagunas Andinas, Planta Piloto de Procesos Industriales y Microbiológicos, Centro Científico Tecnológico, CONICETTucumán, Argentina; ^2^Facultad de Ciencias Naturales e Instituto Miguel Lillo, Universidad Nacional de TucumánTucumán, Argentina; ^3^Centro Integral de Microscopía Electrónica, Universidad Nacional de Tucumán, Centro Científico Tecnológico, CONICETTucumán, Argentina; ^4^CONICET Centro de Excelencia en Productos y Procesos de la Provincia de CórdobaCórdoba, Argentina; ^5^Facultad de Química e Ingeniería, Pontificia Universidad Católica ArgentinaRosario, Argentina; ^6^Instituto de Física Rosario, CONICET Universidad Nacional de RosarioRosario, Argentina; ^7^Facultad Regional Concepción del Uruguay, Universidad Tecnológica NacionalConcepción del Uruguay, Argentina; ^8^Facultad de Ciencias Exactas, Ingeniería y Agrimensura, Universidad Nacional de RosarioRosario, Argentina

**Keywords:** extremophiles, central andes, genomes, astrobiology, microbe, microbialites, stromatolites

## Abstract

The Central Andes region displays unexplored ecosystems of shallow lakes and salt flats at mean altitudes of 3700 m. Being isolated and hostile, these so-called “High-Altitude Andean Lakes” (HAAL) are pristine and have been exposed to little human influence. HAAL proved to be a rich source of microbes showing interesting adaptations to life in extreme settings (poly-extremophiles) such as alkalinity, high concentrations of arsenic and dissolved salts, intense dryness, large daily ambient thermal amplitude, and extreme solar radiation levels. This work reviews HAAL microbiodiversity, taking into account different microbial niches, such as plankton, benthos, microbial mats and microbialites. The modern stromatolites and other microbialites discovered recently at HAAL are highlighted, as they provide unique modern—though quite imperfect—analogs of environments proxy for an earlier time in Earth's history (volcanic setting and profuse hydrothermal activity, low atmospheric O_2_ pressure, thin ozone layer and high UV exposure). Likewise, we stress the importance of HAAL microbes as model poly-extremophiles in the study of the molecular mechanisms underlying their resistance ability against UV and toxic or deleterious chemicals using genome mining and functional genomics. In future research directions, it will be necessary to exploit the full potential of HAAL poly-extremophiles in terms of their biotechnological applications. Current projects heading this way have yielded detailed molecular information and functional proof on novel extremoenzymes: i.e., DNA repair enzymes and arsenic efflux pumps for which medical and bioremediation applications, respectively, are envisaged. But still, much effort is required to unravel novel functions for this and other molecules that dwell in a unique biological treasure despite its being hidden high up, in the remote Andes.

## Introduction

**Figure 1:**
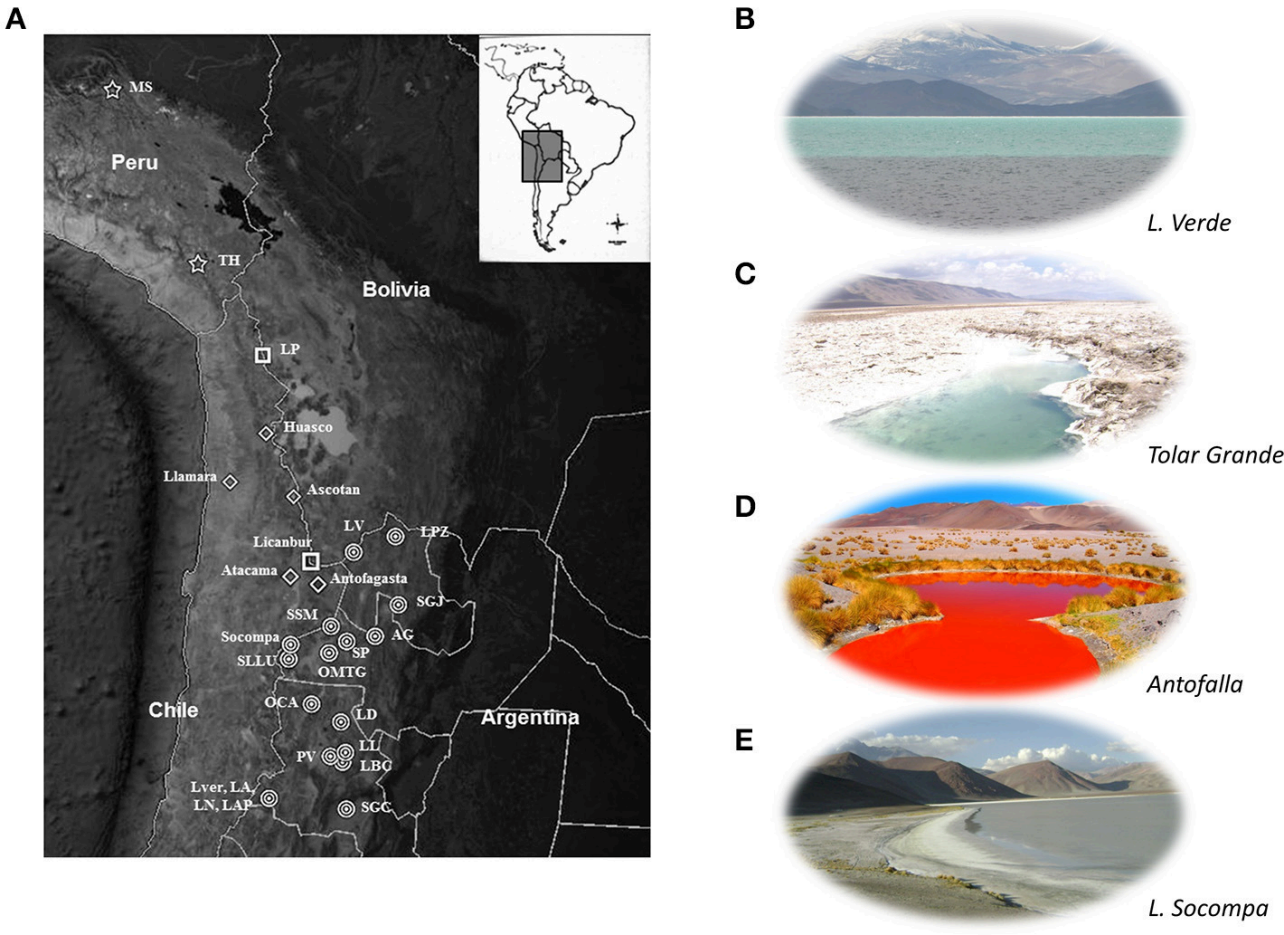
**The HAAL ecosystem. (A)** Geographical location of HAAL (modified from Google Earth). Names are abbreviated as shown on **Table 1**. Locations not abbreviated include more than one lake from the table. Symbols indicate country of origin: Stars: Peru; Squares: Bolivia; diamonds: Chile; circles: Argentina. **(B–E)**: Typical landscape of selected HAAL. **(B)** Lake Verde, Catamarca (4100 m); **(C)** Ojos de Mar Tolar Grande, Salta (3600 m); **(D)** Ojos de Campo Antofalla (3350 m); **(E)** Laguna Socompa (3570 m).

High-Altitude Andean Lakes (HAAL; Figure 1), locally called “Lagunas” (L) or “Salares” (S), are distributed through Argentina, Chile, Bolivia, and Peru along the Central Andes region in South America. These shallow lakes and wetlands are found at up to 6000 m altitude through diverse, extreme ecosystems: (i) the Altiplano or Puna of Atacama, a volcanic upthrusted plateau above 3000 m, (ii) the Atacama Desert, one of the oldest deserts on Earth extending from 20 to 30°S along the Pacific coast of South America and (iii) the western Andean flank between ca. 18°S and 27°S (4000–6000 m) (Chong, 1984; Hartley and Chong, 2002; McKay et al., 2003; Hartley et al., 2005; Cáceres et al., 2007; Escudero et al., 2007; Demergasso et al., 2008; Farías et al., 2009; Ordoñez et al., 2009; Placzek et al., 2009; Albarracín et al., 2011; Wierzchos et al., 2011). In spite of the severe environmental conditions, a flourishing microbial diversity of extremophiles thrives in plankton, benthos, microbial mats, and even microbialites (Zúñiga et al., 1991; Dorador et al., 2003, 2008a,b, 2010, 2013; Demergasso et al., 2004, 2007, 2008, 2010; Ferrero et al., 2004; Fernández Zenoff et al., 2006; Maturrano et al., 2006; Zenoff et al., 2006; Cabrol et al., 2007, 2009; Escudero et al., 2007; Dib et al., 2008, 2009; Farías et al., 2009, 2011a, 2013; Flores et al., 2009; Ordoñez et al., 2009; Belluscio, 2010; Thiel et al., 2010; Lara et al., 2012).

The aim of this work is to review the diversity and biology of extremophilic microbial communities found across diverse niches in these wetlands and which bear great astrobiological and biotechnological importance.

## Environmental background

HAAL are defined mostly on the basis of shared features, in this case geographic location and altitude (Figure 1). Their location and some of the most prominent physico-chemical features affecting the ecology of these aquatic microbial “hotspots” are listed in Table 1.

**Table 1:** **Geographical data and main physico-chemical parameters of HAAL where microbial communities were reviewed in the literature**.

**Location**	**Site**	**Acronym**	**Global position**		**Altitude (masl)**	**pH**	**Arsenic (mg L^−1^)**	**Salinity (ppt)**	**T (°C)**	**References**
Argentina	Laguna Aparejos	LAP	27°39′58.71″S	68°23′26.10″W	4200	6.5	2.5	N.D	N.D	Ordoñez et al., 2009
	Laguna Negra	LN	27°39′20.17″S	68°33′46.18″W	4100	5	3	Hypersaline	15	
	Laguna Verde	LVE	27°34′44.38″S	68°38′48.94″W	4100	6.5	0.8	Hypersaline	14	
	Laguna Azul	LA	27°34′12.67″S	68°32′6.95″W	4450	7.5	0.8	Oligosaline	N.D	
	Laguna Diamante	LD	26°0′49.75″S	67°2′10.08″W	4570	11	230	Hypersaline	14	Rascovan et al., 2015
	Ojos de Campo Antofalla	OCA	25°39′48.96″S	67°42′53.91″W	3350	8.5	N.D	Hypersaline	18	Farias et al., unpublished
	La Lagunita	LL	26°36′S	67°14″W	3800	6.3	N.D	Hypersaline	16	de Mitrovich et al., 2005
	Pasto Ventura	PV	26°43′S	67°10″W	3746	8.1	N.D	Mesosaline	12	
	Blanca	LBC	26°50′S	66°57″W	3250	8.5	N.D	Oligosaline	19	
	Salinas Grandes	SGC	27°48′S	66°48″W	3300	8.3	N.D	Hypersaline	14	
	Laguna Vilama	LV	22°36′10.66″S	66°55′20.78″W	4500	7.1	11.8	Hypersaline	N.D	(Ordoñez et al., 2009)
	Salina Grande	SGJ	23°37′22.88″S	65°53′4.08″W	3400	N.D	N.D	Hypersaline	N.D	
	Laguna Pozuelos	LPZ	22°15′44.28″S	66°01?07.3″W	3600	8.5	N.D	Oligosaline	9	Fernández Zenoff et al., 2006
	Abra del Gallo	AG	24°16′S	66°22″W	4400	8.5	N.D	Oligosaline	11	de Mitrovich et al., 2005
	Laguna Socompa	LS	24°32′10.60″S	68°12′32.64″W	3600	9	33.5	Hypersaline	14	Farías et al., 2011a
	Ojo de Mar Tolar Grande	OMTG	24°37′36.21″S	67°22′25.45″W	3510	6.5	0.59	Hypersaline	14	Farias et al., unpublished
	Salar Santa Maria	SSM	24°5′31.73″S	67°21′16.71″W	4250	11	N.D	Hypersaline	21	
	Salar Llullaillaco	SLLU	24°45′54.54″S	68°15′30.01″W	3750	8.5	N.D	Hypersaline	15	
	Salar Pocitos	SP	24°23′11.56″S	66°59′31.48″W	3660	8	N.D	Hypersaline	19	
Chile: Atacama Desert	Tebenquiche	LT	23°7′50.76″S	68°14′40.68″W	2350	7.08	1.11	Hypersaline	24.2	Dorador et al., 2009; Thiel et al., 2010; Lara et al., 2012
	Burro Muerto	LBM	23°17′14.03″S	68°33′58.64″W	2350	7.8	1.38	Hypersaline	9.6	
	Cejar	LCE	23°3′34.28″S	68°12′44.77″W	2343	7.47	9.62	Hypersaline	18.5	
	Laguna de la Piedra	LDLP	23°3′25.40″S	68°13′5.26″W	2341	7.5	N.D	Hypersaline	25	Stivaletta et al., 2011
Chile: Altiplano Chileno	J1 Pond	SASJ1	21°37′2.77″S	68°14′49.64″W	3744	8.3	3.4	Oligosaline	0	Lara et al., 2012
	LT Pond	SASLT	21°36′20.55″S	68°18′9.84″W	3741	8	1.6	Oligosaline	18	
	V10 Spring	SASV10	21°36′22.3″S	68°14′54.68″W	3740	N.D	0.9	Freshwater	16	
	V6 Spring	SASV6	21°29′42.45″S	68°15′17.66″W	3738	8.3	28	Polysaline	3	
	H0 Spring	SH0	20°15′45.95″S	68°52′31.61″W	3800	7.6	N.D	Mesosaline	17.2	Dorador et al., 2008a,b, 2009
	H1 Pond	SH1	20°16′20.54″S	68°52′45.82″W	3800	8.8	N.D	Mesosaline	17.9	
	H4 Pond	SH4	20°16′24.49″S	68°53′6.16″W	3800	8.5	N.D	Hypersaline	12.3	
	H6 Pond	SH	20°19′33.74″S	68°51′20.08″W	3800	8.6	N.D	Hypersaline	4.9	
	Simba	LSI	23°21′47″S	67°40′55″W	5870	4.7	1	N.D	−1−6.2	Escudero et al., 2007
	Lejía	LLE	23°30′00″S	67°42′00″W	4325	6.9	N.D	Hypersaline	3–10.6	Demergasso et al., 2010
	Salar de Aguas Calientes	SAC	23°07′00″S	67°25′00″W	4200	6.9	N.D	Hypersaline	8–18	
Bolivia/ Chile	Poquentica	LP	18°44′05″S	62°58′28″W	5750	8.4–6.9	0.03	Mesosaline	4.3–14	Cabrol et al., 2007, 2009; Escudero et al., 2007; Demergasso et al., 2010; Fleming and Prufert-Bebout, 2010
	Licanbur	LL	22°50.03′S	67°53.00″W	5916	5	N.D	N.D	4	
Bolivia	Thermales hot spring	THS	22°46.96′S	67°48.15″W	4328	8	N.D	Oligosaline	36.2	
	Laguna Blanca Cold Spring	LBCS	22°48.32′S	67°46.34″W	4340	7.3	N.D	Oligosaline	17.7	
	Blanca	LBL	22°47.00′S	67°47.00″W	4340	7.2–8.42	N.D	Polysaline	14	
	Verde	LVC	22°47.32′S	67°49.16″W	4332	9–8.19	N.D	Hypersaline	12.9	
Peru	Maras salterns	MS	13°18′10″S	72°09′2″W	3380	6.5–7.0	–	Hypersaline	< 20	Maturrano et al., 2006
	Huaytire^a^	TH	16°54′S	70°20″W	4452	6.6 (5.1–7.6)	–	Freshwater	8.7 (7.0–13.4)	Salazar-Torres et al., 2012

a Values are the median for 13 locations. Maximum and minimum are shown in parentheses.

ND, not determined.

**Figure 2:**
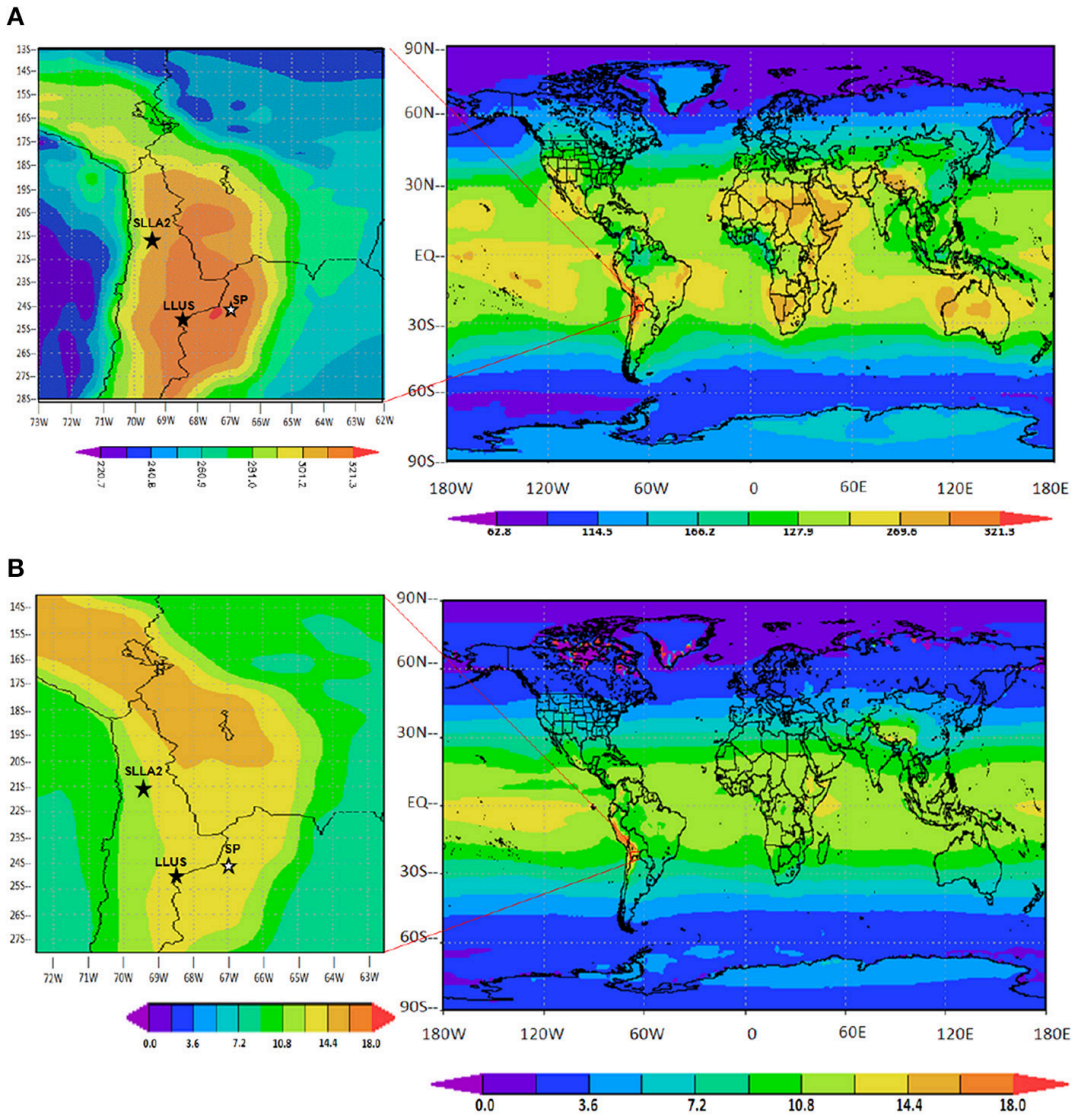
**(A)** Mean daily total solar irradiance (in W m^−2^), adapted from NASA MERRA Monthly History Data Collections (2D); **(B)** Mean local noontime UV Index over the Earth's surface (adapted from NASAOMI/Aura Online Visualization and Analysis, Daily Level 3 Global Gridded Products) http://gdata1.sci.gsfc.nasa.gov, with the region under study highlighted in the left map. Data were obtained by model calculations of spectral solar irradiances employing the SMARTS (version 2.9.5) algorithm developed by Gueymard (1995) together with NASA satellite measurements of climatic variables on selected DCA microbial hotspots (Table 1): i.e., Llullaillaco salar, named LLUS in the map, (6034 m), S. Llamará, Puquio2, named SLLA2 in the map, (800 m) and S. Pocitos, named SP in the map, (3660 m). Since the temperature variation with altitude is about −0.65°C each 100 m of increase (http://eo.ucar.edu/webweather/basic5.html), the indetermination due to the difference in altitude is insignificant.

The climate and meteorology of the Andes in general, and of the Central Andes in particular, present extreme characteristics due to their strong altitude gradients and their meridional distribution, acting as a barrier that affects and determines great part of the environmental conditions along the Southern Hemisphere (Vuille et al., 2000; Vuille and Keimig, 2004; Garreaud et al., 2009). The Central Andes and especially the Puna-Altiplano, register the highest surface solar radiation levels worldwide (Figure 2A), with extreme values of ca. 310 W m^−2^. Monthly average of daily insolation reaches 6.6 kW h m^−2^ d^−1^, a value that is within the highest in the world (Duffie and Beckman, 2013). Cede et al. (2002) and Luccini et al. (2006), described monthly average noon UV Index near the summer solstice above 19 and erythemal daily doses above 10 kJ m^−2^ during December-January. These astonishingly high levels of solar total and UV irradiation were evident at “microbial hotspots” where micro-biodiversity was documented (Table 1): S. Llullaillaco (6034 m), S. Llamará, Puquio 2 (800 m), and S. Pocitos at an intermediate altitude of 3660 m (Figures 2A,B). Many factors contribute to this outcome; tropical latitude gives small noon solar zenith angles throughout the year, while very clean high altitude atmospheres with small aerosol content and scarce cloud amount reduce atmospheric attenuation to solar UV radiation (Luccini et al., 2006). Moreover, the total vertical column of atmospheric ozone typically has its lower normal values over the equatorial and tropical regions, with less variability with respect to mid-latitudes and Polar Regions (Bais et al., 2007). In addition, high albedos due to permanent or occasional snowed surfaces enhance solar radiation intensity on the surface.

**Figure 3:**
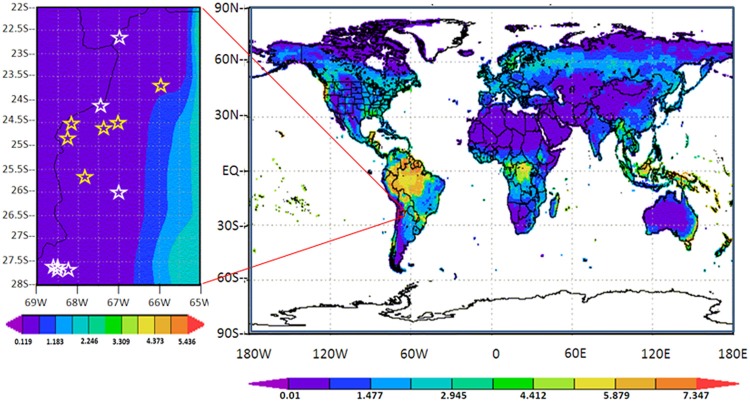
**Leaf Area Index (LAI) (in m^2^ m^−2^) in the decade 2000–2009 for all the Earth (right) and on the Puna of Atacama region (left) with LAI in the range of (0.39 ± 0.27) m^2^ m^−2^**. For this graphic selected HAAL were highlighted (Table 1): i.e., lakes situated at altitudes equal or greater than 4000 m (L_high_): LAP, LN, LVE, LA, LD, LV, and SSM (white stars) and lower than 4000 m (L_low_): OCA, SG, LS, OMTG, SLLU, and SP (yellow stars). Adapted from NASA MERRA Monthly History Data Collections (2D) http://gdata1.sci.gsfc.nasa.gov/.

**Figure 4:**
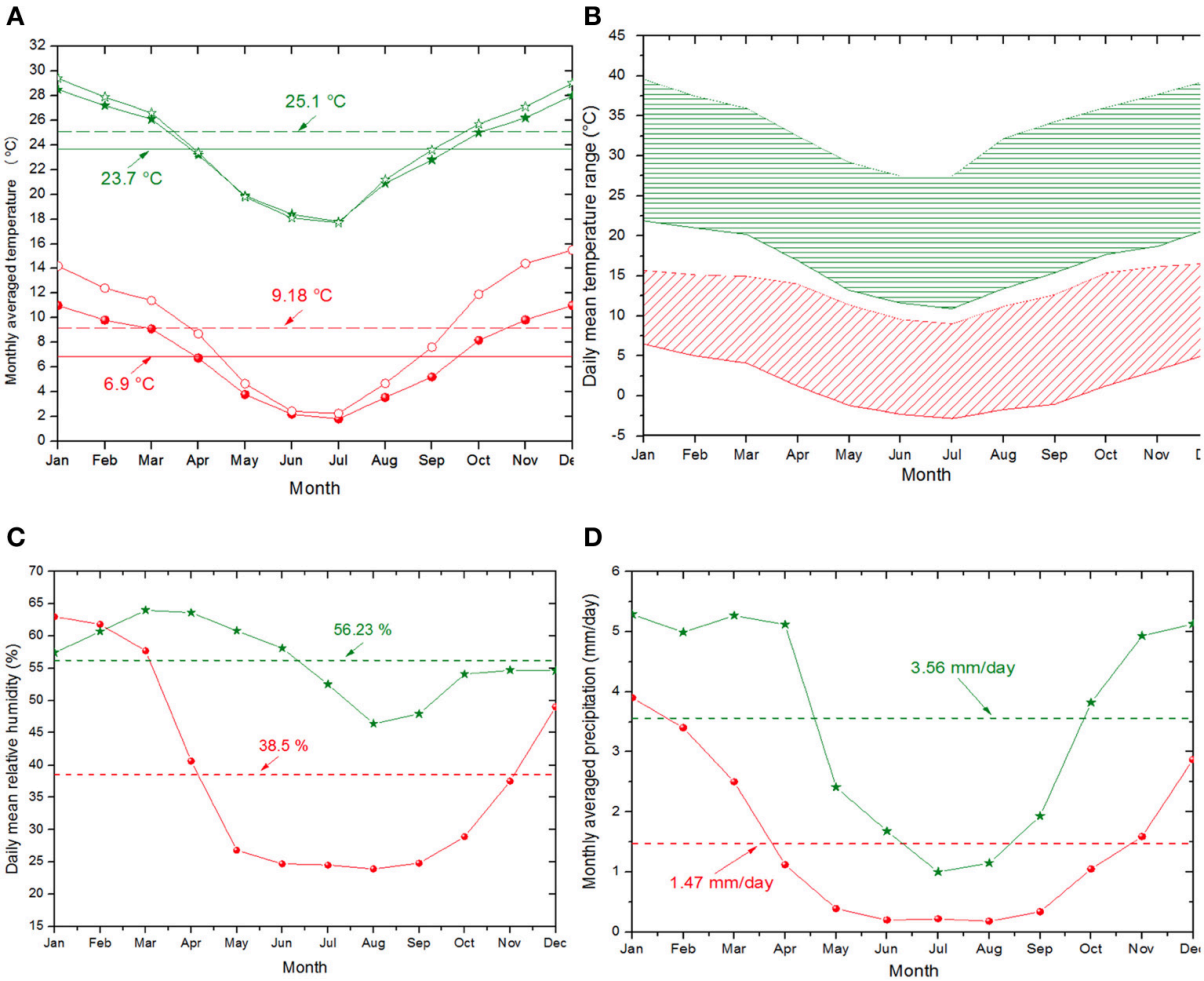
**(A)** Monthly averaged air temperature at 2 m above the surface (solid symbols) and skin temperature (open symbols). The skin temperature is of interest since the microorganisms are normally fixed to soil surface. **(B)** Daily temperature range for S. Pocitos hotspot (in red) and low-altitude site (in green). **(C)** Daily mean relative humidity. **(D)** Monthly averaged precipitation. In both figures, red dots denote S. Pocitos hotspot (3360 m) and green stars to a low-altitude site (87 m) in a similar latitudinal region for comparison. Horizontal lines denote the annual mean values for each case. Adapted from SSE/NASA (http://eosweb.larc.nasa.gov/sse).

The high-altitude and the desert or semi-desert characteristics also impose rigorous meteorological conditions over the region. As clear evidence of the degree of aridity on HAAL's area, Figure 3 shows a global map of Leaf Area Index (LAI), in which the LAI value is one of the smallest worldwide, in the range of 0.39 ± 0.27 m^2^ m^−2^. Accordingly, the central-east strip of the Puna region within the Central Andes is cataloged as an “arid cold steppe” (code BSk), while the west strip is cataloged as an “arid cold desert” (code BWk) (Peel et al., 2007). Yearly mean values of 38.5% and 1.47 mm for relative humidity and precipitation, respectively, are shown for the representative microbial hotspot of S. Pocitos (Figures 4C,D). Mean daily relative humidity (Figure 4C) and monthly precipitation (Figure 4D) display maximum values from January to April, a period called the “Bolivian winter” which produces larger humidity, cloud coverage, and monsoon-like precipitations.

Annual mean air temperature values reach 6.9°C at S. Pocitos in comparison with low-altitude areas at the same inter-tropical latitude where mean air temperature reaches 23.7°C (Figure 4A). In addition, daily temperature fluctuation for this particular hotspot varies between ca. −2.5 and 12.5°C in Jun-Jul-Aug and between ca. 5 and 15°C in Nov-Dec-Jan, indicating that microbes face low temperatures all year round (Figure 4B). Nevertheless, temperature in some lakes such as the L. Licancabur and L. Socompa is much more temperate as abundant hydrothermal seeps pour in these aquatic systems. Licancabur lies within the crater of the Licancabur volcano at 5200 m, where multiple hydrothermal inputs maintain water in its liquid state at 4°C (Cabrol et al., 2007). A hypothesis held about the case of Socompa (3570 m), is that a hydrothermal seep contributes to sustain a regular temperature at the lake area where stromatolites developed (Farías et al., 2013).

At the lakes themselves, salt contents, pH, and arsenic concentration (Table 1) can reach extreme values, generating niches where extremophilic microorganisms develop. Salinity varies greatly among the wetlands (Risacher et al., 2003), some water sources can have a notoriously low salt content while others constitute actual brines (Table 1). Variations are also evident within a same system (Dorador et al., 2003); the Salar de Huasco located at 3800 m in the Chilean Altiplano displays high spatial heterogeneity, represented by a mosaic of streams, *bofedales* (peatlands), shallow permanent and non-permanent lagoons and salt crusts, with a gradient in salt concentration from north to south (Dorador et al., 2008b). Meanwhile in the Vilama area (4500 m), in Argentina, at least nine lakes ranging between slightly saline and hypersaline were described (Derlindati, 1998).

The system pH also influences the distribution of microbial species due to interactions with other components such as sulfate or borate (Oremland et al., 2005). For HAAL, pH can also vary different settings, and this depends on the chemical constituents (Table 1). The presence of carbonates and calcium is coincident with alkaline conditions. Many HAAL are alkaline or slightly alkaline, and there are only a few exceptions where acidic conditions were found (Table 1). No alkaline brines are known in Chilean salt flats because of the higher sulfate and lower alkalinity of inflowing waters as a consequence of the suspected higher sulfur content in Chilean volcanic rocks (Risacher and Fritz, 2009). In salt flats, pH is higher in winter than in summer, with values between 7.0 and 8.5. In certain locations pH values fall off this range, which could be related to the existence of specific minerals present in volcanic settings.

Arsenic (As) by itself can greatly influence the development of life. It is a ubiquitous toxic metalloid released in the environment mainly by volcanic activity (Smedley and Kinniburgh, 2002). The most common oxidation states for soluble arsenic in nature are the pentavalent, arsenate [As(V)], and the trivalent arsenite [As(III)], present as (AsO_4−3_) and (As(OH)_3_), respectively, (Rosen, 2002; Anderson and Cook, 2004)—As(III) being much more toxic than As(V) (Mukhopadhyay, 2002; Rosen, 2002; Oremland and Stolz, 2003). Even though HAAL are pristine environments, they display a high concentration of arsenic in the water due to natural geochemical phenomena and active volcanism (Flynn et al., 2002; Smedley and Kinniburgh, 2002; Mantelli et al., 2003; Romero et al., 2003; Escudero et al., 2007; Dib et al., 2008; Escalante et al., 2009; Ordoñez et al., 2009). Endogenous and exogenous agents, such as meteorological effects on the soil, and the circulation of subterranean water and wind, distribute arsenic through the atmosphere, soil and water (Lara et al., 2012). In several HAAL, As concentrations range between 0.8 mg L^−1^ and 11.8 mg L^−1^ (Table 1). But in L. Socompa (Farías et al., 2013), Salar of Ascotan (Demergasso et al., 2007) and L. Diamante (Rascovan et al., 2015), values in water reach up to 33.8, 183 mg L^−1^, and 230 mg L^−1^, respectively. In S. Ascotan, arsenic concentrations in brine sediments range between 610 and 9440 mg kg^−1^ (Escudero et al., 2013). Such arsenic accumulation significantly exceeds the maximum amount accepted by the World Health Organization (10 μg L^−1^) and is also greater than the one reported for Lake Mono (200 μM ${\text{HAsO}}_{4}^{-\text{2}}$, approximately 19.8 mg L^−1^ As^0^), a hypersaline and alkaline water body (Oremland et al., 2005) in which indigenous microbes and their interaction with As have been widely studied (Wolfe-Simon et al., 2011; Reaves et al., 2012). All these different variable combinations generate a plethora of niches to colonize, giving rise to the surprising biodiversity described in the following sections.

## Microbial diversity

HAAL harbor a distinct and diverse microbiodiversity thriving in most niches: in superficial waters (plankton), colonizing sediments (benthos), or forming cooperative benthonic structures such as microbial mats and microbialites. Diversity is highly dependent on the microecosystem considered, which is briefly reviewed in the next sections. The results presented here are biased toward prokaryotic diversity, although some results on eukaryotic diversity are also revised.

### Plankton/benthos

HAAL are usually shallow lakes (10–400 cm) where benthic communities share members with planktonic communities, as winds might often stir up the sediment (Cabrol et al., 2007). For example, chlorophyll a was much higher in the shallow lake Burro Muerto, where resuspension from the sediments is an important factor (Demergasso et al., 2008). In water samples from L. Blanca and L. Verde, near the Licanbur volcano, diatoms living on the sediment float on the water as tychoplanktonic (adapted to living in still water) elements as a result of wind activity (Cabrol et al., 2007).

Diverse approaches were used to analyze bacterial diversity using culture-dependent and culture-independent techniques such as DGGE (Denaturing Gradient Gel Electrophoresis) (Demergasso et al., 2004; Escudero et al., 2007) and rRNA gene clone libraries (Demergasso et al., 2008; Dorador et al., 2008a,b, 2013). Demergasso et al. (2008) reported predominance of Bacteroidetes and Gammaproteobacteria in L. Tebenquiche while in athalassohaline lakes of the Atacama Desert, Bacteroidetes phylum and a few Proteobacteria were the main taxa (Demergasso et al., 2004). Seventy-eight phylotypes of Cyanobacteria were identified in water and sediment samples from Salar de Huasco, with Oscillatoriales, Pleurocapsales, Chroococcales, and Nostocales as the main taxonomical groups (Dorador et al., 2008b). Dorador et al. (2009) explored bacteroidetes diversity in both water and sediment samples from S. de Ascotan, S. de Huasco and S. de Atacama (L. Tebenquiche). Cluster analysis (WPGMA) of DGGE bands showed that bands from S. de Huasco and S. de Ascotan grouped together. Samples from S. de Atacama formed separate clusters in water and sediment samples, reflecting different bacteroidetes communities between the geographically separated locations. Demergasso et al. (2010) sampled water and sediment from Laguna Lejía, Salar de Aguas Calientes, and a lake at the Simba volcano summit. The microbial community structures at Salar de Aguas Calientes and Laguna Lejía were similar to those from other saline systems and cold environments where Bacteroidetes is the major bacterial group. Dorador et al. (2013) constructed clone libraries using rRNA gene sequences for bacteria from sediment and water. Most of the sequences were affiliated within Bacteroidetes, Proteobacteria, Firmicutes, Actinobacteria, Verrucomicrobia, Deinococcus-Thermus, Planctomycetes, Acidobacteria, Cyanobacteria, Chloroflexi, Gemmatimonadetes, and the Candidate division WS3.

Bacterial diversity on the Argentinean HAAL was quite similar to the one found in Chilean S. of Ascotan, S. of Huasco, L. Licanbur, L. Aguas Calientes, L. Chungará, Parinacota wetland and L. Piakota (Demergasso et al., 2004, 2007, 2008, 2010; Escudero et al., 2007; Lara et al., 2012; Dorador et al., 2013). Proteobacteria, Firmicutes, Actinobacteria and Bacteroidetes were the dominant bacterial taxa of the planktonic community of L. Pozuelos, L. Azul, L. Vilama, L. Negra, L. Aparejos and L. Verde (Ferrero et al., 2004; Fernández Zenoff et al., 2006; Zenoff et al., 2006; Dib et al., 2008; Flores et al., 2009; Ordoñez et al., 2009). Interestingly, microbial community composition was highly variable not only among lakes, but also between water and sediment samples from the same site and among different sampling points within the same lake (Demergasso et al., 2004). Heterogeneity has also been observed in water samples from L. Tebenquiche (Demergasso et al., 2008), which has been attributed to salinity and to chemical and temperature gradients. This is also expected to occur in benthos, although it has not yet been investigated. Another source of variation in benthos would be the differences in seasonal water levels, as many sites underwater in the wet season become exposed in the dry season, with considerable differences in the physicochemical conditions. In Maras Salterns, in the Peruvian Andes (Maturrano et al., 2006), bacteria were also represented by Proteobacteria, Firmicutes and Bacteroidetes, which confirms the prevalence of these three groups at HAAL.

A remarkable aspect of HAAL microbiodiversity is that, in general, bacterial communities dominate planktonic microecosystems—with Archaea represented to a much lower extent (Escudero et al., 2007; Demergasso et al., 2010; Dorador et al., 2013; Rascovan et al., 2015). Depending on the salt content of the basins, Archaea were characterized by sequences related to halophilic Euryarchaeota (Halobacteria), non**-**halophilic Euryarchaeota, methanogens Euryarcheota, and mesophilic Crenarchaeota in Chile (Demergasso et al., 2004; Dorador et al., 2013) but only halophilic Euryarcheota were found in the Argentinean HAAL (Rascovan et al., 2015) and in the Peruvian Maras Salterns (Maturrano et al., 2006).

Apart from prokaryotic diversity, HAAL plankton also bears a rich and unique eukaryotic community. Studies of South American high-altitude aquatic systems were usually taxonomic and sometimes aimed at paleolimnology. In general, diatoms have been reported as the main algal group (Sylvestre et al., 2001; Tapia et al., 2003, 2006). Other floristic studies of systems located between 2420 and 4683 m (Maidana and Seeligmann, 2006; Seeligmann, 2008; Maidana et al., 2011), including not only HAAL, but also rivers and freshwater wetlands in northwestern Argentina, analyzed the taxonomy of diatoms in the area. The genera *Nitzschia* and *Navicula* were the most abundant and more widely distributed. In the Lipez region in Bolivia *Navicula* was also highly represented (Álvarez-Blanco et al., 2011). Taxonomic affiliations to the species level are difficult; the Andes region presents numerous endemisms comprising a characteristic flora (Metzeltin and Lange-Bertalot, 2007; Rivera and Cruces, 2009) with new species described in several of these reports (Díaz and Maidana, 2006; Blanco et al., 2013). Taxonomic classifications are complicated, as some of the local studies (in Spanish) might not be available to or acknowledged by foreign researchers, prompting reports on taxa that have already been described.

Another study performed in the Huaytire wetland, in Peru, analyzed variation along several seasons of the year and areas with and without human impact (Salazar-Torres et al., 2012). Diatoms were the most abundant algal group, making up 95% of the total abundance while Chlorophyceans and cyanobacteria contributed only to 3 and 2% of the total, respectively. Significant differences were found in the total microalgae abundance among the three periods and areas. In comparison to the ice-covered and ice-free seasons, abundance was remarkably higher during the ice-melting season. Higher abundance was also significant in the livestock area.

Abundance and diversity strongly depend on the physicochemical conditions of the lakes. The best example would be the lakes at the base of the Licanbur volcano (Cabrol et al., 2007): in L. Blanca (LBL), the high Si concentration in the water favors diatom biomass development. The phytoplankton in LBL is poor in euplanktonic elements, both in species and individual numbers. The number of living cells is extremely high: 82,000 ind/ml. Though connected to LBL, L.Verde (LVC) has an extremely different physicochemical environment (see Table 1), with different depths and salinities. Only one euplanktonic species (*Gymnodinium* sp.) was found in the samples collected with plankton nets. All other species are tychoplanktonic diatoms. The individual number of algae is low (91 ind/ml), rendering the lake oligotrophic. It has been speculated that the relative absence of euplanktonic species might be due to the strong UV pressure on these very high lakes, but given the differences in chemical setting, this would be difficult to prove. The proportion of teratological diatom frustules in LBL was found to be 1–2% (ten times higher than normal), which might be an effect of UV radiation. Nevertheless, much species diversity was encountered for thycoplanctonic diatomea (Cabrol et al., 2007).

The Licancabur summit lake (LL, 5916 m) is one of the highest on Earth (Cabrol et al., 2007). In water samples from LL, poor diversity and abundance was observed: one Cyanobacteria, three Chrysophyceae, one Euglenophyta, two Chlorophyceae species. Among zooplankton, two Ciliata, and several Hyphomyceta species were found. These observations pointed out to the presence of a food web (primary producers, consumers, decomposers). Further data indicated the presence of more dense populations of zooplankton at LL, LBC, and S. de Aguas Calientes (Cabrol et al., 2009). At LL, the study of one-off zooplankton samples from the lake revealed the presence of novel species of testate amoeba (De Smet and Gibson, 2009).

The relation with environmental variables was carefully explored in diatom communities from thirteen lakes in southwest Bolivia (Servant-Vildary and Roux, 1990; Servant-Vildary et al., 2000). One hundred and seven species were found and their optima and tolerance to the ionic composition of the waters (anions and cations), alkalinity, salinity, depth, pH, and density were obtained. Diatom assemblages were linked to ionic elements rather than to salinity, pH, depth, temperature or elevation. This was later applied to reconstruct models of past lake water salinity and ionic concentration in the southern Bolivian Altiplano (Sylvestre et al., 2001).

### Microbial mats and microbialites

Mats are multilayered, multidimensional matrixed microbial communities that incorporate detritus, minerals and associated geochemical materials, including crystals (Brigmon et al., 2008). The interwoven patterns can form laminated or concentric structures (Noffke et al., 2001). Microbialites are organosedimentary structures accreted by sediment trapping, binding and *in situ* precipitation due to the growth and metabolic activities of benthic microbial mat communities. Stromatolites and thrombolites are morphological types of microbialites classified by their internal mesostructure: layered and clotted, respectively, (Burne and Moore, 1987). Microbialites first appeared in geological records 3.5 billion years ago, and for more than 2 billion years they were the main evidence of life on Earth (Desnues et al., 2008). Microbialites are somewhat restricted today, often due to competition with other types of organisms—mainly the heterotrophic eukaryotes that evolved more recently. If present, modern analogs are found mostly in ecologically stressed environments such as hypersaline lagoons, coastal zones, thermal springs, and alkaline lakes (Dravis, 1983; Reid et al., 2000; Burns et al., 2004; Desnues et al., 2008; Andersen et al., 2011; Berelson et al., 2011).

**Figure 5:**
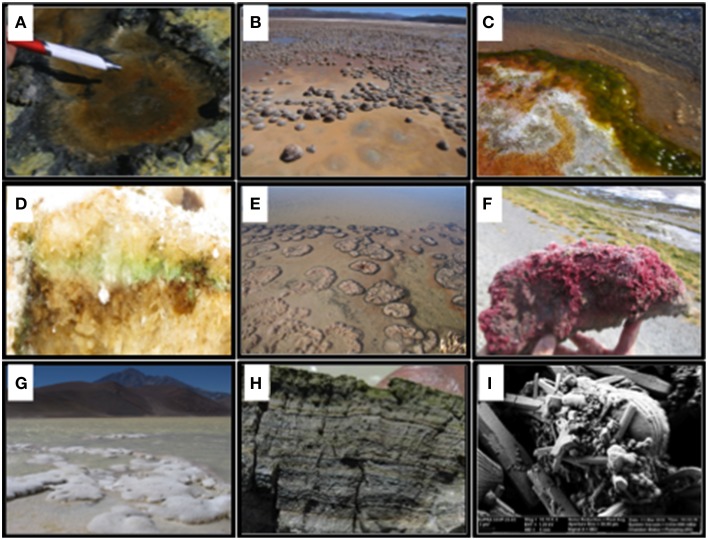
**Cooperative microbial communities at the HAAL. (A)** Microbial mat in L. Vilama; **(B)** L. Negra; **(C)** Hydrothermal vent with mat near L. Socompa; **(D)** Evaporitic Mat at S. de Llamará; **(E)** L. Tebenquiche (Salar de Atacama); **(F)** Microbialite from L. Diamante; **(G)** L. Socompa; **(H)** Socompa stromatolite vertical section; **(I)** Scanning Electron Microscopy (SEM) image of Socompa stromatolites, showing diatoms and crystals agglutinated by organic structures.

In geology, the study of modern stromatolites helped to identify the factors operating and considered today in the interpretation of ancient samples (Margulis et al., 1986). Thus, their search and study is critical since they provide *in-hand* models for inferring possible evolutionary scenarios for the beginning of life on Earth. Modern stromatolites (Belluscio, 2009; Farías et al., 2011a, 2013) and other microbialites were discovered recently at HAAL (Figure 5) (Farías et al., 2014; Rasuk et al., 2014, 2015; Rascovan et al., 2015) and provide unique modern—though quite imperfect—analogs of environments proxy for an earlier time in Earth's history (Belluscio, 2009). The elements that help support this assertion are the volcanic setting and profuse hydrothermal activity, low atmospheric O_2_ pressure, thin ozone layer, and high UV exposure (Demergasso et al., 2003; Cabrol et al., 2007).

Microbial mats were first described at the salt flat Salar de Llamará, Atacama Desert, Northern Chile (Demergasso et al., 2003). The authors described three different types of mats where predominant microorganisms varied according to the mat layer; i.e., oxygenic phototrophic layers formed by diatoms, unicellular cyanobacteria (*Cyanothece* and *Synechococcus* spp.) and filamentous cyanobacteria (*Microcoleus* sp. and *Oscillatoria* sp.), while anoxygenic phototrophic layers where formed by *Chromatium* and *Thiocapsa* spp. Also in the Salar de Llamará, a recent study by Rasuk et al. (2014) described a special case of lithified microbial mats as gypsum dome-shaped bioherms. The domes were found partially submerged in water and their microbial diversity was shown to be season-dependent (winter/summer). Its diversity comprises mainly Proteobacteria (Alphaproteobacteria and Gammaproteobacteria), Bacteroidetes and Verrucomicrobia. Stivaletta et al. (2011) studied the endolithic microbial communities from gypsum domes at Laguna de la Piedra (2341 m). In this case, they found Cyanobacteria, Bacteroidetes, Alphaproteobacteria, Betaproteobacteria, and Deltaproteobacteria halophilic archaea, ciliates and members of the candidate division TM6. Gypsum-dominated microbialites and domes were observed at Laguna Tebenquiche (2350 m) and calcium carbonate-containing mats and microbialites were detected at Laguna La Brava (Farías et al., 2014). Mats and microbialites were much more diverse than gypsum domes. Bacteroidetes were dominant microbes in Tebenquiche while Proteobacteria prevailed in La Brava mats. Diatoms and cyanobacteria were observed under the electron microscope, but the latter were below 1% in the 16S pyrotags, stressing the idea that primary production might be done by anoxygenic phototrops.

Thiel et al. (2010) analyzed phototrophic communities of red-purple-colored microbial mats on the sediments surface in L. Tebenquiche and L. Chaxas. Sequence analysis of genes from the photosynthetic machinery revealed high heterogeneity among the anoxygenic phototrophic bacterial communities and possibly the presence of yet unknown phototrops within the different samples of both lakes (Thiel et al., 2010). The communities had a significantly different composition in the two lakes as well as in subsamples of each of the lakes. Changing conditions such as water variability, salt concentrations and light apparently shaped the community structure in the microhabitats of salty lakes. Based on previous studies, biological productivity in these lakes was expected to be high (Boschetti et al., 2007). However, the content of chlorophyll *a* was shown to be rather low (Demergasso et al., 2008), leading to the assumption that there was a considerable impact of anoxygenic phototrophic bacteria on the primary productivity in such habitats. For this study, functional genes were used as molecular targets to specifically analyze anoxygenic phototrophic bacteria. Phylogenetic congruence between the ribosomal genes and genes for specific structural components of the photosynthetic apparatus of green sulfur bacteria (fmoA, Alexander et al., 2002; Alexander and Imhoff, 2006) and of phototrophic purple bacteria (*pufLM*, Tank et al., 2009) confirmed the presence of such organisms. *pufLM* genes were successfully amplified from 10 out of 11 samples, while *fmoA* genes were not amplified from any environmental sample. Nevertheless, enrichment cultures did amplify this marker, which might suggest that a low number of organisms are present in the environmental samples. The high number of as-yet-uncultured and unidentified *pufLM* phylotypes retrieved emphasizes the uniqueness of the studied area as well as the need for further studies on phototrophic bacteria, including culture-dependent approaches. Fleming and Prufert-Bebout (2010) characterized the cyanobacterial composition of a variety of microbial mats present in three lake systems: L. Blanca, L. Verde (4300 m), a summit lake in the Licancabur Volcano cone (5970 m), and a few adjacent geothermal springs. These communities contained many heterocytous, nitrogen-fixing cyanobacteria (e.g., *Calothrix, Nostoc, Nodularia*) as well as a large number of cyanobacteria belonging to *Leptolyngbya*. More than a third (37%) of all taxa in this study were new species (96% 16S rRNA gene sequence identity), and 11% represented new and novel taxa distantly related (93% identity) to any known cyanobacteria. There was very little compositional overlap between the different sample sites at L. Blanca, L. Verde, and the Licancabur summit lake. It is probable that high environmental heterogeneity in the area has led to the segregation of populations. This finding conforms to the presence of a variety of mat morphologies.

Mats and sediments sampled from different hypersaline lakes L. Cejar, L. Llamara, L. Jachucoposa, and Laguna Pujsa located in salt flats of the Atacama Desert were subjected to massive sequencing of the V4 region of the 16S rRNA genes of Bacteria. Diversity was higher in sediment than in mats and mats were observed always at higher saline lakes. Proteobacteria and Bacteroidetes were again the major taxa represented in all samples (Rasuk et al., 2015).

The most studied HAAL stromatolites are those from L. Socompa (3570 m), located at the southern shore of the lake lying at the bottom of the active Socompa Volcano (Figure 5G) (Farías et al., 2013). The water were stromatolites develop is alkaline, hypersaline, rich in inorganic nutrients, very rich in arsenic, and warm (20–24°C) due to a hydrothermal input. Forming broad, rounded domes, their composition is dominated by diatom frustules and aragonite micro-crystals agglutinated by extracellular substances (Figure 5I). Biodiversity analysis by 454 pyrosequencing of the amplified V4 region of 16S rRNA gene showed that the microbial community harbors highly abundant representatives of *Deinococcus-Thermus*, Rhodobacteraceae, Desulfobacterales, and Spirochaetes, and a surprisingly low abundance of Cyanobacteria compared to the other modern stromatolites. Sequences that could not be classified at phylum level showed less than 80% identity to the best hit in the NCBI database, suggesting the presence of novel distant lineages. The primary production in the stromatolites was generally high and dominated by *Microcoleus* sp. Diatoms—other important oxygenic photosynthetic organisms in HAAL environments—were dominated by *Amphora* sp. Surprisingly, they were abundant in the anoxic, sulfidic and essentially dark parts of the stromatolites and were found mainly as naked and crushed frustules, suggesting that they must be only sediment material within the stromatolite rather than metabolically active diatoms.

Other fascinating systems at HAAL are located at a hypersaline shallow lake (Laguna Diamante) within the Galán volcano (Rascovan et al., 2015). Partially submerged calcareous formations (microbialites) thrived outstanding chemical hamper including pH up to 11 and poisoning arsenic concentrations (115–234 mg/L). Interestingly, their bottom part had red biofilms (DLRB) composed of round microbial cells and gaylussite crystals embedded in a matrix of extracellular polymeric substances (Rascovan et al., 2015). Most of the DLRB was formed by Archaea, mainly *Halorubrum* with a minor proportion (6%) of Bacteria belonging to anaerobic Clostridia and Chromatiales. The metabolic potential of these biofilms was explored specially for arsenic metabolism; Rascovan et al. (2015) hypothesized that DLRB employ different metabolic strategies in response to environmental changes. As oxygen is limited, these microorganisms might adopt anaerobic respiration on different substrates but mainly arsenate, which at the same time would provide energy and promote biofilm growth.

### Model poly-extremophiles

**Figure 6:**
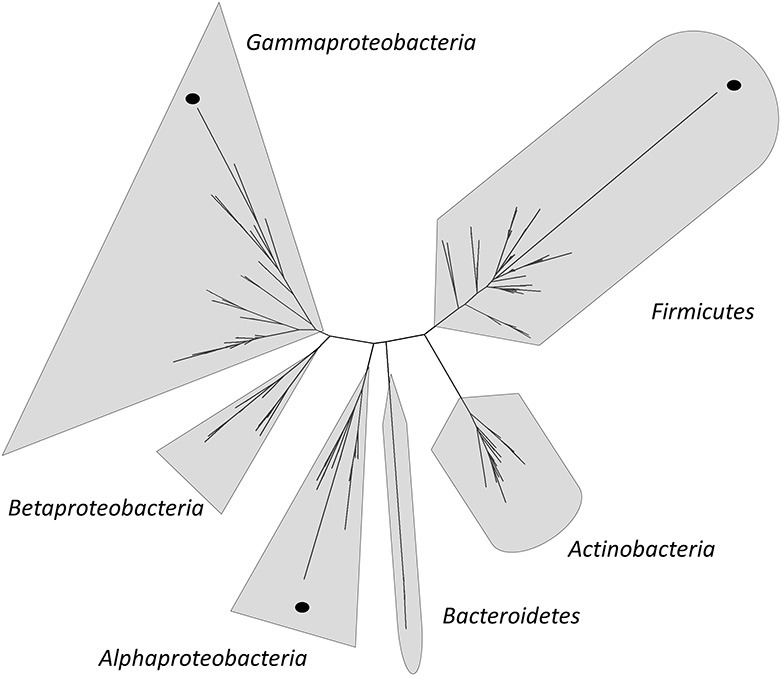
**Phylogenetic tree from 138 16S rRNA gene partial sequences from HAAL strains and 202 type strains from RDP (Supplementary File S1)**. The tree was built using methods implemented in QIIME 1.5.0 (Caporaso et al., 2010b). The sequences were aligned using PyNAST aligner (Caporaso et al., 2010a) and the model 16S rRNA genes Greengenes alignment (McDonald et al., 2012). The alignment was filtered and a maximum likelihood tree was constructed using Fasttree (Price et al., 2010). Sequences with low homology to type strains are highlighted by a dot.

In the last years, several authors have contributed to the sampling of water, sediments, soils, flamingo feces, microbial mats, and microbialites from diverse locations at HAAL (Table 1) (Dorador et al., 2003, 2008a,b, 2010, 2013; Demergasso et al., 2004, 2007, 2008, 2010; Fernández Zenoff et al., 2006; Zenoff et al., 2006; Cabrol et al., 2007, 2009; Escudero et al., 2007; Dib et al., 2008, 2009; Farías et al., 2009; Flores et al., 2009; Ordoñez et al., 2009; Albarracín et al., 2011). Using these samples, strain isolation programs were guided toward specific taxonomic groups, microbial resistances or characteristics, such as their remarkable resistance to high salinity, UV-B irradiation, antibiotics (ATBs), oxidative stress and arsenic levels (Fernández Zenoff et al., 2006; Dib et al., 2008, 2009; Farías et al., 2009; Flores et al., 2009; Ordoñez et al., 2009). Most isolates displayed a combination of extreme resistances; hence, they have been named poly-extremophiles (Albarracín et al., 2011). Ordoñez et al. (2009) reported that the Gammaproteobacteria are the most UV-B-resistant phylogenetic group in Andean lakes. Flores et al. (2009) performed tolerance test on different concentrations of NaCl on the strains isolated from surface water of L. Verde and L. Negra (Table 1). Twelve isolates classified as medium or low salinity tolerant according to their specific growth rate on 1 and 5 % of NaCl-amended media. Only *Pseudomonas* sp. N23 and *Pseudoalteromonas* sp. N32—both isolated from L. Negra—were able to grow in media amended with 10% NaCl. On the other hand, isolated bacteria from HAAL with arsenic resistance phenotypes were described by Dib et al. (2008). In this study, arsenite resistance was found in 8 of the 13 tested strains which were able to grow on synthetic media amended with up to 5 mM As(III). Some strains were even able to grow well on 10 mM As(III): *Acinetobacter johnsonii (*A2), *Rhodococcus erithropolis* (V2), *Micrococcus* sp. (V7), *Staphylococcus saprophyticus* (A3), and *Brachybacterium* sp. (V5). Most Eubacteria and Archaea isolates from HAAL have been analyzed by 16S rRNA gene sequencing, and partial sequences have been deposited in GenBank. A subset of 138 of these sequences with lengths above 800 bp was used to build a phylogenetic tree (Figure 6) together with sequences of 202 type strains obtained from the Ribosomal Database Project^1^ (Supplementary File S1). Most strains are close relatives to type strains, however, a few remarkable isolates distant from their closest relatives can be seen highlighted with black dots.

HAAL model poly-extremophiles were later used for studying molecular mechanisms underlying their resistance ability against UV and toxic or deleterious chemicals; *Sphingomonas* sp. S17 isolated from the modern stromatolite community of L. Socompa was the first genome sequence reported from the HAAL ecosystem (Farías et al., 2011b). Consistent with the extreme environment and high UV irradiation, *Sphingomonas* sp. S17 presented a complete DNA repair system, including indirect and direct photodamage repair systems, neither of which was found in the reference genome of *Sphingomonas wittichii* RW1. Accordingly, this bacterial genome also presented 24 genes involved in sulfur metabolism, compared to only 4 such genes in *S. wittichii*. A set of 95 genes was present in the subsystem for resistance to antibiotics and toxic compounds compared to only 41 such genes in *S. wittichii*. The majority of these genes are devoted to the resistance of arsenic, chromium, and fluoroquinolones, with 18 genes devoted to multidrug resistance efflux pumps. The genome also contained two copies of a NhaA-type CDS for the Na^+^/H^+^antiporter and six subunits of the multisubunit cation antiporter (Na^+^/H^+^) compatible with its alkaline and hypersaline environment.

Through a proteomic approach, Belfiore et al. (2013) described the proteins involved in the arsenic resistance process of another model extremophile isolated from L. Socompa, stromatolite *Exiguobacterium* sp. S17. The comparative analysis of S17, exposed and unexposed to arsenic—As(V) and As(III)—revealed 25 proteins differentially expressed. Under arsenic stress, proteins involved in energy-metabolism, stress, transport, and in protein synthesis were noticeably up-regulated. The genome sequence of *Exiguobacterium* sp. S17 was likewise obtained, emphasizing the fact that it contains a complete DNA repair system, including UvrABC, MutL-MutS, and bacterial photolyase, and several genes related to toxic compound resistance, such as antibiotics, arsenic, cadmium, and mercury (Ordonez et al., 2013). Genomic comparisons with available public genomes from *Exiguobacterium* suggests that one of the reasons behind high resistance to arsenic in S17 is the presence of the *acr3* gene, a known contributor to cell detoxification against arsenite, one of the most toxic arsenic species (Ordoñez et al., 2015). All *Exiguobacterium* strains have a conserved *arsB* gene playing a role similar to *acr3*, but S17 is the only one bearing both genes.

The mechanisms of extreme UV resistance have been analyzed in *Acinetobacter* sp. Ver3, isolated from L. Verde, through genomic and proteomic analysis (Kurth et al., 2015). In addition to early data indicating its role of catalases and photolyases in resistance (Di Capua et al., 2011; Albarracín et al., 2012), a “UV-resistome” was defined which encompasses genes related to this phenotype, including putative regulators such as a novel cryptochrome and a LuxR type regulator, DNA repair/maintenance systems, error-prone DNA polymerases, and genes related to oxidative stress (Kurth et al., 2015). Diverse mechanisms, including aminoacid and protein synthesis, were observed to be up-regulated under UV-B exposure, while proteins related to several energy-generating pathways such as glycolysis, beta-oxidation of fatty acids and electronic respiratory chains were down-regulated, as revealed by proteomics. Thus, novel targets interesting for further characterization were identified in this study.

Three novel arsenic resistant strains of *Salinivibrio* spp. (up to 200 mM As), NaCl (up to 15%), and UV-B radiation (19 kJ/m^2^) isolated from different niches of L. Socompa were also used as model systems for the study of molecular mechanisms involved in poly-resistance profiles (Gorriti et al., 2014). Among many putative systems related with multiple-stress resilience, the three draft genomes displayed genes related with osmotic stress, DNA repair, arsenic detoxification systems, hydrolytic enzymes, ferredoxins, flavodoxins, proteorhodopsins, and xanthorhodopsins. With respect to DNA repair, the authors described genes involved in the recombinational and nucleotide excision repair, including RecBCD helicase/nuclease and UvrABC endonuclease holoenzymes. Homologs for *recA* and *recX* genes (SOS response) were also detected in the genomes of strains S34 and S35. In addition, they presented gene homologs to deoxyribodipyrimidine photolyase, and one gene coding for a transcriptional regulator of the Mer family, associated with photolyases.

## Concluding remarks and future perspectives

In this paper, we have reviewed work on the (micro)biological diversity of these unique ecosystems. Given the extension and altitude ranges at HAAL, certain variation could be expected on the climatological and physicochemical parameters. However, these have been shown to be consistently extreme all along the whole Puna-Altiplano region, with values as high as a monthly mean UV Index above 19, UV erythemal daily doses above 10 kJ m^−2^, total solar irradiances close to the solar constant, daily total horizontal insolation of about 6.6 kW h m^−2^, intense dryness and large daily ambient thermal amplitude, together with the exposure to a multitude of chemicals and physical stresses, including alkalinity, high concentrations of arsenic and dissolved salts, and dust and ashes from volcanic activity. This combination of hostile factors makes HAAL a modern Earth-based analog to Mars and has been an important target of a wide number of astrobiological expeditions in the last decades. Moreover, as modern stromatolites were found thriving in L. Socompa we proposed these systems as model analogs of their Precambrian-counterparts giving way to renewed studies on how life evolves on Earth (stromatolites) and, potentially, elsewhere. Also of astrobiological interest are the poly-extremophilic microbes herein described which offers diverse model systems for the study of evolutionary processes, enzyme stabilization and activation, under extreme conditions.

HAAL ecosystems constitute natural laboratories for exploring and monitoring *in situ* interactions between the geophysical background and the dynamics of biodiversity. Aridity might be the main factor limiting life in the region, and that is why diverse microbial communities are associated to water. On the lakes themselves, solar irradiation (including high UV-B doses) is without a doubt a common factor putting great pressure on the ecology of microbial communities, even though the diverse physicochemical conditions generate a wide plethora of niches where extremophilic microorganisms develop. Salinity is another main factor that may be limiting diversity. The different combinations of factors make HAAL, some of whose lakes host complex ecosystems, including zooplankton, heterogeneous.

Microenvironment heterogeneity correlates with several levels of microbial diversity. This has been assessed by both, culture-dependent and independent approaches, obtaining isolates from many HAAL and using DGGE, clone libraries and 16S V4 hypervariable region pyrosequencing. Bacteroidetes, Firmicutes, and Proteobacteria (mainly Alphaproteobacteria and Gammaproteobacteria) were found to be the major bacterial taxonomical groups developing in Chilean and Argentinean HAAL. Cyanobacteria and eukaryotic micro-algae played an important role as key constituents of microbial mats and microbialites rather than as planktonic elements, a clear reflection of UV impact on planktonic communities. It is considered that Cyanobacteria are fundamental to microbial mats and sediments as primary producers and as determinants of the organomineralization producing exopolisaccharide (EPS). However, in most HAAL mats, cyanobacterial OTUs were scarce and even absent. This is more evident in mats, microbialites, and evaporites in the Atacama Desert, where McKay et al. (2003) suggested that Cyanobacteria might not be a dominant phylum (Azua-Bustos et al., 2012). They measured the moisture under the stones and observed that it was not enough for Cyanobacteria life. Several reports indicated that primary production, usually performed by Cyanobacteria, could be partly substituted by other organisms such as diatoms or nonphototrophic carbon fixers, since dark carbon fixation by chemoautotrophic bacteria might be a big contributor to the overall carbon fixation, especially in sediments with low organic matter content. But still, little is known about the importance of this process in lake systems, despite the assumption that there is high chemoautotrophic potential in lake sediments.

Euryarcheota representatives were found widely distributed in the salt flats and lakes but as a rule, Archaea appeared always in much lower proportions than bacteria did for a fixed sampling point or location. This finding may be due to bias in the methodology used or in the sampling process or perhaps because of low efforts applied in the isolation of such hard-to-cultivate microbes. More scientific efforts need to be placed in this direction.

Besides diatoms, eukaryotic diversity has been scarcely studied at HAAL as they tend to be less diverse and less represented on extreme environments. However, they are major players in the ecosystem of several HAAL, for example as a food resource for flamingo species. Diatoms might have a role also as primary producers in microbial mats while Fungi are likely producers of interesting secondary metabolites. More research on the roles and identities of eukaryotic microorganisms are required.

HAAL exhibit a distinct and diverse microbiodiversity thriving in all niches. Nevertheless, data on each niche is unequally produced with much descriptive work available on plankton and benthos of alkaline and neutral lakes. Less attention was paid to the microbial communities of acidic lakes, fumaroles or hydrothermal vents, microbial mats, and stromatolites. As mentioned earlier, studies on diversity have revealed the complexity and uniqueness of both mats and microbialites. The interplay of its members and their role in the community as well as in the shaping of the environment by precipitation of minerals constitute another path within the research. We have only briefly discussed recent findings on HAAL communities, but geochemical and microbiological studies in the area will expand the knowledge on the subject.

In future research directions, it will be necessary to exploit the full potential of HAAL poly-extremophiles in terms of their biotechnological applications (Albarracín and Farías, 2012), such as the production of waxes and fatty acids for biodiesel (Bequer Urbano et al., 2013), or compatible solutes, antioxidants, pigments, or enzymes for the pharmaceutical industry (Farías et al., 2011b). Current projects heading this way have yielded detailed molecular information and functional proof on novel extremoenzymes: i.e., photolyase of *Acinetobacter* sp. Ver3 (Albarracín et al., 2014), and an arsenical resistance efflux pump from *Exiguobacterium* sp. S17 (Ordoñez et al., 2015) for which medical and bioremediation applications, respectively, are envisaged. But still, much effort is required to unravel novel functions for this and other molecules that dwell in a unique biological treasure despite its being hidden high up, in the remote Andes.

## Author contributions

VA conceived, organized, and wrote the paper. DK, VA, CB, OO, EL, GS, RP recollected data, analyzed the existing information and contributed with the writing. MF had the original project idea, performed expeditions, sampled the HAAL, provide strains and genomes and revised the manuscript. MF, RP, and VA obtained funding for the original project idea. All authors have read and approved this manuscript.

### Conflict of interest statement

The authors declare that the research was conducted in the absence of any commercial or financial relationships that could be construed as a potential conflict of interest.

## Footnotes

^1^RDP, http://rdp.cme.msu.edu/.

## Supplementary material

Supplementary File S1 **Subset of 138 of 16S rRNA gene sequences with lengths above 800 bp used to build a phylogenetic tree, in order to highlight potentially rare strains together with sequences of 202 type strains obtained from Ribosomal Database Project (RDP, http://rdp.cme.msu.edu/)**.
